# Neurocognitive Status after Aortic Valve Replacement: Differences between TAVI and Surgery

**DOI:** 10.3390/jcm10081789

**Published:** 2021-04-20

**Authors:** Nicholas Aroney, Tiffany Patterson, Christopher Allen, Simon Redwood, Bernard Prendergast

**Affiliations:** Cardiovascular Directorate, Guy’s and St Thomas’ NHS Foundation Trust, London SE1 7EH, UK; nicholas.aroney@gmail.com (N.A.); tiffany.patterson@gstt.nhs.uk (T.P.); christopher.allen@gstt.nhs.uk (C.A.); simon.redwood@gstt.nhs.uk (S.R.)

**Keywords:** TAVI, surgery, SAVR, stroke, neurocognitive

## Abstract

Over the past decade, indications for transcatheter aortic valve implantation (TAVI) have progressed rapidly—procedural numbers now exceed those of surgical aortic valve replacement (SAVR) in many countries, and TAVI is now a realistic and attractive alternative to SAVR in low-risk patients. Neurocognitive outcomes after TAVI and SAVR remain an issue and sit firmly under the spotlight as TAVI moves into low-risk cohorts. Cognitive decline and stroke carry a significant burden and predict future functional decline, reduced mobility, poor quality of life and increased mortality. Early TAVI trials used varying neurocognitive definitions, and outcomes differed significantly as a result. Recent international consensus statements defining endpoints following TAVI and SAVR have standardised neurological outcomes and facilitate interpretation and comparison between trials. The latest TAVI and SAVR trials have demonstrated more consistent and favourable neurocognitive outcomes for TAVI patients, and cerebral embolic protection devices offer the prospect of further refinement and improvement.

## 1. Background

Over the past decade, indications for transcatheter aortic valve implantation (TAVI) have progressed rapidly—procedural numbers now exceed those of surgical aortic valve replacement (SAVR) in many countries, and TAVI is now a realistic and attractive alternative to SAVR in low-risk patients. Neurocognitive outcomes after TAVI and SAVR remain an issue and sit firmly under the spotlight as TAVI moves into low-risk cohorts. Cognitive decline and stroke carry a significant burden and predict future functional decline, reduced mobility, poor quality of life and increased mortality [1,2]. Early trials suggested that TAVI may have a significant stroke penalty, potentially prohibiting its use in lower-risk patients [3]. On the contrary, alongside improvements in TAVI technology and increased operator experience, recent trials have shown that TAVI may improve neurocognition and cause less stroke, and that previously reported high stroke rates were likely due to increased ascertainment by specialist neurologists [4,5].

The original large Society of Thoracic Surgeon (STS) studies found low levels of stroke (1.5% and 3.7%) after valve surgery, but did not incorporate a prespecified neurological review [6,7]. More recent studies that included neurological adjudication and cerebral imaging have demonstrated central nervous system (CNS) infarcts on cerebral magnetic resonance imaging (MRI) in up to 61% of patients after SAVR, with clinical stroke in 17% (and more likely with larger lesion volumes) [2].

**Figure 1:**
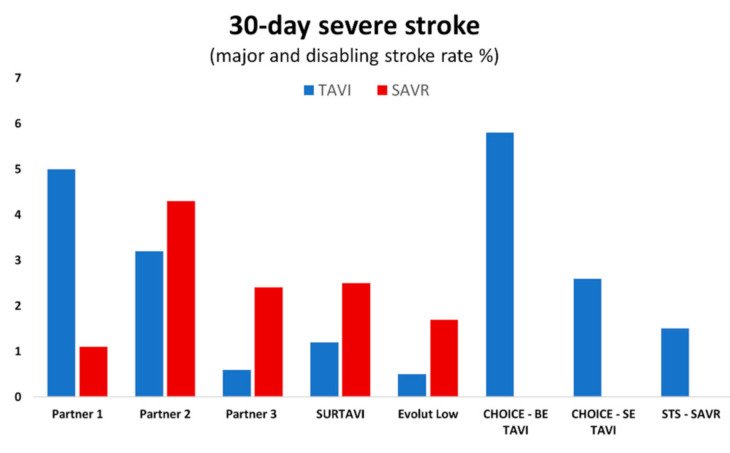
Rates of major and disabling stroke at 30-day follow-up in pivotal trials for TAVI (transcatheter aortic valve implantation) and SAVR (surgical aortic valve replacement).

**Figure 2:**
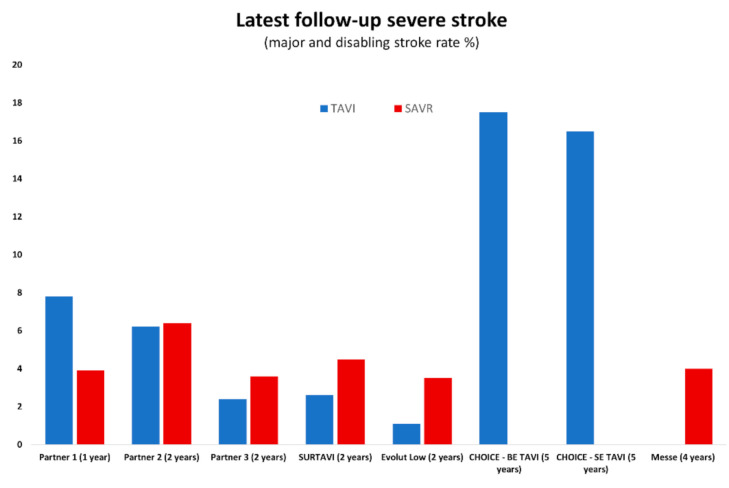
Rates of major and disabling stroke in pivotal trials at most recent follow-up (years from procedure).

Rates of stroke in the pivotal TAVI trials vary from 1.6–5.9% [8,9] as a likely consequence of differing definitions and mechanisms of adjudication (Figure 1 and Figure 2). In initial trials, cardiologists adjudicated neurological events without access to specialist neurological review or cerebral imaging. More recent TAVI studies have identified new brain lesions on an MRI in 98% of patients, and these lesions (even silent infarcts) may be associated with adverse neurological events [10,11].

Three consortia have provided guidelines for endpoint definitions following TAVI and SAVR in an attempt to standardise neurological outcomes and facilitate interpretation and comparison between trials—the Valve Academic Research Consortium (VARC-2), American Heart Association/Stroke Association (AHA/ASA), and Neurologic Academic Research Consortium (NeuroARC) (Table 1) [12,13,14]. The most recent NeuroARC consortium developed a framework to assess, measure and classify procedure- and device-related neurological endpoints in a three-stage classification (Type 1—overt CNS injury, Type 2—covert CNS injury, and Type 3—neurologic dysfunction without CNS injury) [13]. NeuroARC aims to provide definitions for reproducible endpoints, classification of neurological events, and methods for consistent event identification, adjudication and reporting [13].

## 2. Neurocognition: Cardiac Surgery

Neurocognitive dysfunction after cardiac surgery has been recognised since the 1960s, with more recent studies showing it can affect 30–70% of patients [15,16,17,18]. Whilst patient-related risk factors (such as age, prior cognitive function, frailty, alcohol abuse, and depression) are primarily non-modifiable [19], procedure-related risks (including the use of cardiac bypass, thermoregulation, procedural time, anaesthetic dose, and regulation of blood pressure and glycaemic control) may be potentially mitigated [19]. Several strategies have been developed to prevent and treat post-operative neurocognitive dysfunction, with only modest benefits [19], and more recent studies have shown more pronounced impairment in neurocognition following SAVR compared with coronary artery bypass grafting [20], suggesting that more extensive injury may occur in patients with aortic stenosis as a result of liberated particulate matter and microemboli at the time of surgery [20,21]. Furthermore, although one study demonstrated that 47% of patients have new ischaemic brain lesions on a cerebral MRI, these were not associated with neurocognitive decline at discharge [22].

## 3. Neurocognition: TAVI vs. SAVR

Previous studies of high-risk patients have demonstrated elevated incidence of stroke, higher NIHSS (National Institute of Health Stroke Scale) scores and a greater fall in MMSE (mini-mental state examination) after SAVR compared to TAVI [23,24]. In one intermediate-risk trial, TAVI was associated with a global improvement in cognitive status compared to SAVR, and this improvement was more pronounced in patients with cognitive impairment pre-TAVI (MMSE ≤ 27) [25]. More post hoc analysis of the PARTNER-3 low-risk population demonstrated cognitive improvements in all those with pre-existing impairment (MMSE ≤ 27) at 30-days, and sustained improvement in the TAVI group at one-year follow-up [26]. A recent meta-analysis assessing cognitive outcomes after TAVI identified no significant change in peri-procedural cognitive performance (<7-days), an improvement at 1 month, but no significant improvement at 6 months or final follow-up [1].

## 4. Stroke: TAVI vs. SAVR in Intermediate-Risk Populations

Two large, randomised trials have shown that TAVI is non-inferior to SAVR in intermediate-risk patients across a variety of endpoints, including all-cause death and disabling stroke at 24 months [27,28]. These trials paid particular attention to neurological outcomes, since patients were a lower-risk cohort than those included in earlier studies. Neurological review thresholds and stroke protocols were similar in both trials, with minor variations in endpoint definitions and follow-up specifications. In PARTNER-2, all patients were reviewed by a trained neurologist, and stroke was defined as a modified Rankin scale score (mRS) ≥ 2 at 90 days after the index clinical event-all strokes were adjudicated by a specialist stroke neurologist blinded to procedural details. In SURTAVI, assessment was undertaken by a specialist neurologist and disabling stroke defined according to VARC-2 criteria (mRS ≥ 2 with an increase by at least one mRS category at 90 days)-NIHSS was measured at baseline, 30 and 90 days in those with events, and cerebral imaging was recommended [12,29,30]. The PARTNER-2 trial found no difference in the incidence of disabling stroke between TAVI and SAVR at 30 days (3.2% vs. 4.3%) or 2-year follow-up (6.2% vs. 6.4%) (Table 2) [28]. Similarly, SURTAVI demonstrated non-inferiority of TAVI compared to SAVR with respect to disabling stroke at 30 days (1.2% vs. 2.5%) and 24 months (2.6% vs. 4.5%) (Table 2). In an ensuing neurological sub-study, there were numerically fewer disabling strokes after TAVI at 30 days (1.2% vs. 2.4%) and 1 year (2.1% vs. 3.3%), though these differences were not statistically significant [31]. Further observations included (A) lower rates of post-procedural encephalopathy after TAVI (1.6% vs. 7.8%; *p* < 0.001); (B) higher overall mortality in patients with early stroke and encephalopathy in both cohorts; and (C) high likelihood of stroke in hypertensive subjects after SAVR [31].

## 5. Stroke: TAVI vs. SAVR in Low-Risk Populations

Initial trials comparing TAVI and SAVR in high and intermediate-risk patients posed important questions regarding stroke and neurological outcomes after TAVI, which might be of even greater clinical significance in younger, low-risk patients [3,27,28]. In parallel, TAVI technology improved considerably, allowing simplified, shorter procedures under conscious sedation using low profile delivery systems.

Reflecting these considerations, the more recent low risk trials included prespecified endpoints and rigorous neurological testing at baseline and follow-up [4,5]. All patients in the low-risk PARTNER-3 trial underwent neurological examination at baseline and 30 days, and those with suspected post-procedural stroke underwent additional NIHSS and mRS at 90 days [5]. Rates of stroke were significantly lower after TAVI than SAVR at 30 days (0.6% vs. 2.4%; *p* = 0.02) and 1 year (1.2% vs. 3.1%; *p* = 0.03), although this difference was no longer apparent at the more recent 2-year follow-up (2.4% vs. 3.6%; *p* = NS) [32].

Although overall stroke rates were similar after TAVI and SAVR at 30-days (3.4% vs. 3.4%; *p* = NS) and 1-year (4.1 vs. 4.3%; *p* = NS) follow-ups in the Evolut Low-Risk trial, the incidence of disabling stroke was significantly lower after TAVI at 30 days (0.5% vs. 1.7%; *p* < 0.05), and remained lower at 2 years (1.1% vs. 3.5%; *p* < 0.05) (Table 2) [4].

Stroke rates are consistently reduced in low-risk compared with intermediate-risk patients as a result of their more favourable clinical risk profile (younger age, less comorbidities), use of new generation TAVI delivery systems, streamlined techniques, and increasing operator experience (Table 2) [4,27,28,32].

## 6. Stroke: Balloon-Expandable vs. Self-Expanding Transcatheter Valves

Despite numerous mechanical and procedural differences between balloon-expandable and self-expanding TAVI devices, comparative studies have demonstrated similar neurological outcomes [29,30]. In the head-to-head CHOICE trial, stroke rates did not differ significantly at 30 days between balloon-expandable and self-expanding valves (5.8% vs. 2.6%; *p* = NS) and 5-year follow-up (17.5% vs. 16.5%; *p* = NS) in high-risk populations [29]. Similarly, a meta-analysis of 35,347 patients (mostly high risk) found no difference in 1-year stroke rates after TAVI using two different device designs (5.0% vs. 4.1%; *p* = NS) [30].

## 7. Neurocognition and Stroke: Alternative Access TAVI and SAVR

Although transfemoral TAVI is preferable for procedural safety and patient comfort, numerous alternative access routes have emerged to allow the procedure in patients with significant peripheral vascular disease. Although minimal access SAVR is also feasible, aortic cross-clamping and aortopulmonary bypass remain necessary. As a consequence, improved clinical outcomes have been clearly demonstrated with transfemoral TAVI, whereas minimal access SAVR has only been associated with reduced duration of hospital stay and overall recovery times [28,33].

The PARTNER-2 sub-group analysis comparing transfemoral and transapical TAVI showed a significantly lower rate of disabling stroke at 30 days in the transfemoral cohort compared with SAVR (2.3% vs. 4.2%; *p* < 0.05), although this difference was not maintained at 2-year follow-up. Rates of disabling stroke were equivalent in the transapical TAVI and SAVR cohorts, with a trend suggesting an increase in non-disabling stroke at 30 days after transapical TAVI [28]. In the UK-TAVI registry, there was no difference in the individual outcome of stroke when comparing four separate treatment groups: SAPIEN transfemoral, SAPIEN transapical, CoreValve transfemoral, and CoreValve subclavian [34]. Furthermore, a large meta-analysis comparing transfemoral and transaxillary TAVI showed no difference in stroke outcomes (3.8% vs. 3.3%; *p* = NS) [35]. Moreover, all-cause mortality was lower after transaxillary TAVI compared with transapical and transaortic access (5.3% vs. 8.4%; *p* < 0.01), albeit with higher rates of stroke in the transaxillary cohort (6.3% vs. 3.1%; *p* < 0.05) [36].

## 8. The Impact of Patient and Procedural Risk Factors

**Figure 3:**
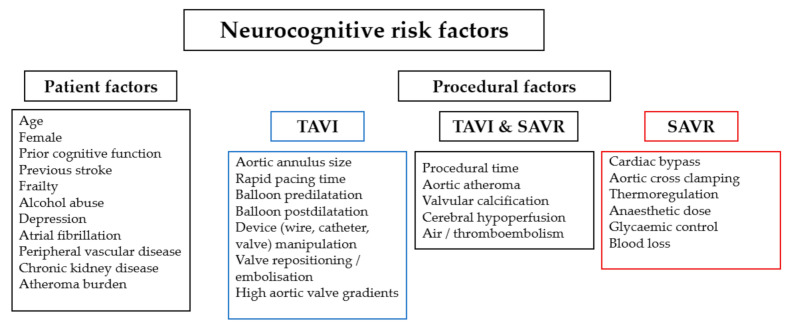
Patient and procedural risk factors for neurocognitive complications.

TAVI and SAVR share numerous patient-related risk factors that increase the likelihood of adverse neurocognitive outcomes, including underlying cognitive dysfunction, age, and renal impairment (Figure 3). Procedure-related risk factors common to both procedures include procedural duration, calcification of the valve and aorta, hypotension, and embolism (Figure 3). Procedure-specific risk factors include aortic cross-clamping, use of cardiopulmonary bypass and blood loss for SAVR, and balloon dilatation, device manipulation and pacing time for TAVI (Figure 3) [17,19,37,38,39].

## 9. Cerebral Embolic Protection

**Figure 4:**
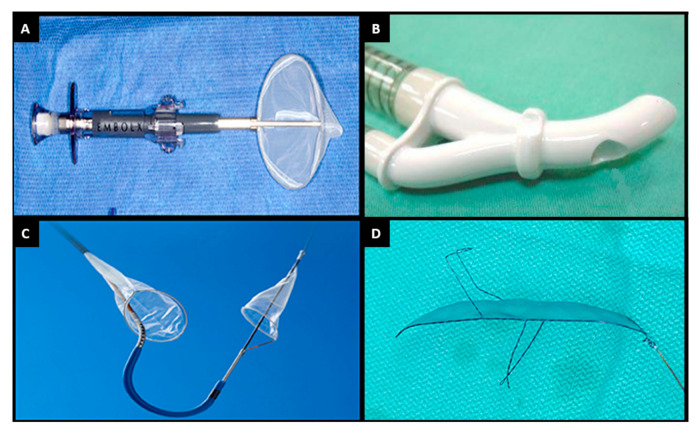
Currently available cerebral embolic protection devices. (**A**) Embol-X. (**B**) Cardio-Gard. (**C**) Sentinel. (**D**) TriGUARD. Adapted with permission from Armijo, G. et al.; *Front. Cardiovasc. Med.*; 2018 [37].

The majority of neurological events following TAVI or SAVR occur within 30 days of the procedure, and result from embolism of thrombotic or calcific material from the native valve leaflets, aortic wall or left ventricular myocardium [40,41]. Quantitative computed tomography assessment has shown that bulky, non-calcific aortic valve tissue is associated with increased major adverse cardiac event (MACCE) rates [42]. Histopathologic analysis of captured debris demonstrated that native bicuspid valves are the highest risk for dislodging large particles, and that valve repositioning is associated with a large quantity of debris [43]. A variety of cerebral embolic protection devices are in development that may reduce the risk of post-procedural neurocognitive dysfunction and stroke (Figure 4).

Two devices, one an intra-aortic filtration system (Embol-X, Edwards Lifesciences, Irvine, CA, USA) and the other a suction-based system (CardioGard, CardioGard Medical, Or-Yehuda, Israel), are approved for use in the United States at the time of SAVR (Figure 4A,B). Large, randomised trials have compared these devices to conventional treatment and, although debris were captured in most cases, the trials were halted prematurely after failing to show any benefit based upon clinical and radiographic endpoints (although rates of in-hospital delirium were significantly lower with suction-based systems) (Table 3) [44]. High stroke rates in the trials were attributed to active ascertainment with neurological assessment and cerebral imaging.

Embolic debris are found in association with most TAVI procedures when using filter-based cerebral protection systems and are more common following device oversizing or the use of a balloon-expandable valve [45]. Initial trials of the Sentinel (Boston Scientific, Marlborough, MA, USA) cerebral embolic protection device—a dual filter system delivered via the right radial artery (Figure 4C)—demonstrated device safety, with non-inferior MACCE rates compared with control subjects (7.3% vs. 9.9%; *p* = NS). Rates of stroke were lower but did not reach statistical significance (5.6% vs. 9.1%, *p* = NS) and, although rates of neurocognition deficit were similar between groups, there was a correlation between decline in neurocognitive function and volume of lesions detected on a cerebral MRI (Table 3) [45]. A subsequent large single-centre propensity-matched study demonstrated a significant reduction in both the primary composite endpoint of all-cause mortality or stroke (2.1% vs. 6.8%; *p* = 0.01) and stroke alone (1.4% vs. 4.6%; *p* = 0.03), associated with cerebral protection [46], whilst a smaller study using a diffusion-weighted MRI to compare TAVI groups with and without cerebral protection reported a numerically smaller volume of cerebral lesions (95 mm^3^ vs. 197 mm^3^; *p* = NS), fewer patients with no new lesions (20% vs. 0%; *p* = 0.03), and less neurocognitive deterioration (4% vs. 27%; *p* = 0.02) in the Sentinel cohort [47]. Furthermore, a large TVT registry showed no significant reduction in in-hospital or 30-day stroke, but suggested a possible relative risk reduction of 20% after a propensity-weighted analysis. Based on these early results, clinical use increased from 7% of TAVI procedures in the United States in 2018 to 28% in 2019. Definitive information concerning safety and efficacy will hopefully be provided by two large-scale randomised controlled trials that are currently underway—BHF-Protect and PROTECTED TAVR [48,49].

TriGUARD is a transfemoral system designed to deflect debris away from all three arch vessels during TAVI (Figure 4D). The early DEFLECT III trial demonstrated that the device was safe, with fewer ischaemic brain lesions on a diffusion-weighted MRI (11.5% vs. 26.9%; *p* < 0.05), reduced neurological deficits detected by NIHSS (3.1% vs. 15.4%; *p* < 0.05), and improved neurocognition compared with control subjects undergoing TAVI without cerebral protection (Table 3) [50]. However, the more recent REFLECT II trial was terminated early due to safety concerns in the active treatment arm, primarily driven by TAVI-related vascular complications [51].

Given the very low stroke rates in low-risk populations, further risk–benefit and cost- effectiveness analyses are required to ascertain which patients derive the most benefit from the use of cerebral embolic protection devices [52].

## 10. Conclusions

Indications for TAVI are expanding into low-risk populations, accompanied by renewed emphasis on the short- and long-term neurological outcomes of the procedure. Streamlined definitions and frameworks, such as the recently published NeuroARC consensus statement, allow clearer for comparison of current and historical trials, and enhance Heart Team decision-making processes. Recent trials have incorporated rigorous specialist neurological input, with a consequent increase in the detection of neurological endpoints after both TAVI and SAVR. Initial concerns that TAVI may be associated with an increased incidence of neurological complications have been allayed, with recent data suggesting improvement in the rates of early stroke and neurocognitive defects. Embolic protection devices offer the prospect of further improvement in current outcomes, and have already demonstrated their effectiveness in reducing surrogate endpoints defined by cerebral imaging (diffusion-weighted MRI)—ongoing large-scale randomised controlled trials are destined to demonstrate meaningful clinical outcomes.

## Figures and Tables

**Table 1:** Stroke definitions provided by international consensus statements.

American Heart Association/American Stroke Association [14]	Valve Academic Research Consortium-2 [12]	NeuroARC [13]
**Definition of CNS infarction:** CNS infarction is brain, spinal cord, or retinal cell death attributable to ischaemia, based on: **1.** Pathological, imaging, or other objective evidence of cerebral, spinal cord, or retinal focal ischaemic injury in a defined vascular distribution; or **2.** Clinical evidence of cerebral, spinal cord, or retinal focal ischaemic injury based on symptoms persisting ≥24 h or until death, and other aetiologies excluded.	Disabling stroke: An mRS score of 2 or more at 90 days and an increase in at least one mRS category from an individual’s pre-stroke baseline.	**Type 1.a Ischaemic stroke:** Sudden onset of neurological signs or symptoms fitting a focal or multifocal vascular territory within the brain, spinal cord, or retina, that: **a.** CNS infarction in the corresponding vascular territory (with or without haemorrhage); or **b.** Absence of other apparent causes (including haemorrhage), even if no evidence of acute ischaemia in the corresponding vascular territory is detected; or **c.** Symptoms lasting <24 h, with pathology or neuroimaging confirmation of CNS infarction in the corresponding vascular territory.
**Definition of ischaemic stroke:** An episode of neurological dysfunction caused by focal cerebral, spinal, or retinal infarction.	**Non-disabling stroke:** An mRS score of <2 at 90 days or one that does not result in an increase in at least one mRS category from an individual’s pre-stroke baseline.	**Type 2.a Covert CNS infarction:** Brain, spinal cord, or retinal cell death attributable to focal or multifocal ischaemia, on the basis of neuroimaging or pathological evidence of CNS infarction, without a history of acute neurological symptoms consistent with the lesion location.
**Definition of silent CNS infarction:** Imaging or neuropathological evidence of CNS infarction, without a history of acute neurological dysfunction attributable to the lesion.	**Stroke:** duration of a focal or global neurological deficit ≥24 h; or 24 h if available neuroimaging documents a new haemorrhage or infarct; or the neurological deficit results in death.	**Type 3.a TIA:** Transient focal neurological signs or symptoms (lasting <24 h) presumed to be due to focal brain, spinal cord, or retinal ischaemia, but without evidence of acute infarction by neuroimaging or pathology (or in the absence of imaging).

Abbreviations: CNS = central nervous system, mRS = modified Rankin scale, TIA = transient ischaemic attack.

**Table 2:** Rates of disabling stroke in intermediate and low-risk trials.

	Intermediate-Risk Patients						Low-Risk Patients					
	PARTNER-2			SURTAVI			PARTNER-3			Evolut Low-Risk		
	TAVI	SAVR		TAVI	SAVR		TAVI	SAVR		TAVI	SAVR	
30 days	3.2%	4.3%	*p* = NS	1.2%	2.5%	*p* = NS	0.0%	0.4%	*p* = NS	0.5%	1.7%	*p* < 0.05
2 years	6.2%	6.4%	*p* = NS	2.6%	4.5%	*p* = NS	0.6%	0.6%	*p* = NS	1.1%	3.5%	*p* < 0.05

**Table 3:** Cerebral embolic protection devices.

	Embol-X^®^	CardioGard^®^	Sentinel^®^	TriGUARD^®^
Manufacturer	Edwards Lifesciences, USA	CardioGard, Israel	Boston Scientific, MA, USA	Keystone Heart, Israel
Filter	Heparin-coated polyester mesh filter; pore size: 120 µm	Suction sideport adjacent to aortic perfusion cannula	Two oval coned mesh filters; pore size: 140 µm	Nitinol frame and mesh filter; pore size: 130 µm
Delivery	Direct aortic cannulation, above cross-clamp	24 Fr direct aortic cannulation	6 Fr radial	9 Fr femoral
Primary mechanism	Filter and capture	Particulate and gaseous suction-based extraction	Filter and capture	Deflection
Coverage	Ascending aorta distal to cross-clamp	Ascending aorta distal to cross-clamp	Brachiocephalic and left common carotid arteries	Brachiocephalic, left common carotid, left subclavian arteries
Pertinent trial	Mack et al. No benefit vs. conventional therapy	Mack et al. No benefit vs. conventional therapy. Lower rates of in-hospital delirium (*p* < 0.05)	SENTINEL-Non-inferior MACCE (*p* = NS) Less stroke numerically but not-significant (*p* = NS)	DEFLECT III-fewer ischaemic brain lesions (*p* < 0.05), reduced neurological deficits on NIHSS (*p* < 0.05), improved neurocognition
Recent/ongoing trials	Nil recruiting	Nil recruiting	BHF-Protect and PROTECTED TAVR	REFLECT II trial was terminated early due to safety concerns

## References

[1] Khan M.M., Herrmann N., Gallagher D., Gandell D., Fremes S.E., Wijeysundera H.C., Radhakrishnan S., Sun Y.R., Lanctôt K.L. (2017). Cognitive Outcomes After Transcatheter Aortic Valve Implantation: A Metaanalysis. J. Am. Geriatr. Soc..

[2] Messé S.R., Acker M.A., Kasner S.E., Fanning M., Giovannetti T., Ratcliffe S.J., Bilello M., Szeto W.Y., Bavaria J.E., Hargrove W.C. (2014). Stroke after aortic valve surgery: Results from a prospective cohort. Circulation.

[3] Smith C.R., Leon M.B., Mack M.J., Miller D.C., Moses J.W., Svensson L.G., Tuzcu E.M., Webb J.G., Fontana G.P., Makkar R.R. (2011). Transcatheter versus Surgical Aortic-Valve Replacement in High-Risk Patients. N. Engl. J. Med..

[4] Popma J.J., Deeb G.M., Yakubov S.J., Mumtaz M., Gada H., O’Hair D., Bajwa T., Heiser J.C., Merhi W., Kleiman N.S. (2019). Transcatheter Aortic-Valve Replacement with a Self-Expanding Valve in Low-Risk Patients. N. Engl. J. Med..

[5] Mack M.J., Leon M.B., Thourani V.H., Makkar R., Kodali S.K., Russo M., Kapadia S.R., Malaisrie S.C., Cohen D.J., Pibarot P. (2019). Transcatheter Aortic-Valve Replacement with a Balloon-Expandable Valve in Low-Risk Patients. N. Engl. J. Med..

[6] O’Brien S.M., Shahian D.M., Filardo G., Ferraris V.A., Haan C.K., Rich J.B., Normand S.-L.T., Delong E.R., Shewan C.M., Dokholyan R.S. (2009). The Society of Thoracic Surgeons 2008 Cardiac Surgery Risk Models: Part 2—Isolated Valve Surgery. Ann. Thorac. Surg..

[7] Shahian D.M., O’Brien S.M., Filardo G., Ferraris V.A., Haan C.K., Rich J.B., Normand S.-L.T., DeLong E.R., Shewan C.M., Dokholyan R.S. (2009). The Society of Thoracic Surgeons 2008 Cardiac Surgery Risk Models: Part 3—Valve Plus Coronary Artery Bypass Grafting Surgery. Ann. Thorac. Surg..

[8] Meredith I.T., Walters D.L., Dumonteil N., Worthley S.G., Tchétché D., Manoharan G., Blackman D.J., Rioufol G., Hildick-Smith D., Whitbourn R.J. (2016). 1-Year Outcomes With the Fully Repositionable and Retrievable Lotus Transcatheter Aortic Replacement Valve in 120 High-Risk Surgical Patients with Severe Aortic Stenosis: Results of the REPRISE II Study. JACC Cardiovasc. Interv..

[9] Kodali S., Thourani V.H., White J., Malaisrie S.C., Lim S., Greason K.L., Williams M., Guerrero M., Eisenhauer A.C., Kapadia S. (2016). Early clinical and echocardiographic outcomes after SAPIEN 3 transcatheter aortic valve replacement in inoperable, high-risk and intermediate-risk patients with aortic stenosis. Eur. Heart J..

[10] Knipp S.C., Matatko N., Wilhelm H., Schlamann M., Thielmann M., Lösch C., Diener H.C., Jakob H. (2008). Cognitive Outcomes Three Years After Coronary Artery Bypass Surgery: Relation to Diffusion-Weighted Magnetic Resonance Imaging. Ann. Thorac. Surg..

[11] Haussig S., Mangner N., Dwyer M.G., Lehmkuhl L., Lücke C., Woitek F., Holzhey D.M., Mohr F.W., Gutberlet M., Zivadinov R. (2016). Effect of a Cerebral Protection Device on Brain Lesions Following Transcatheter Aortic Valve Implantation in Patients with Severe Aortic Stenosis: The CLEAN-TAVI Randomized Clinical Trial. JAMA.

[12] Kappetein A.P., Head S.J., Généreux P., Piazza N., van Mieghem N.M., Blackstone E.H., Brott T.G., Cohen D.J., Cutlip D.E., van Es G.A. (2012). Updated standardized endpoint definitions for transcatheter aortic valve im-plantation: The Valve Academic Research Consortium-2 consensus document (VARC-2). Eur. J. Cardiothorac. Surg..

[13] Lansky A.J., Messé S.R., Brickman A.M., Dwyer M., van der Worp H.B., Lazar R.M., Pietras C.G., Abrams K.J., McFadden E., Petersen N.H. (2017). Proposed Standardized Neurological Endpoints for Cardiovascular Clinical Trials: An Academic Research Consortium Initiative. J. Am. Coll. Cardiol..

[14] Sacco R.L., Kasner S.E., Broderick J.P., Caplan L.R., Connors J.J.B., Culebras A., Elkind M.S.V., George M.G., Hamdan A.D., Higashida R.T. (2013). An Updated Definition of Stroke for the 21st Century. Stroke.

[15] Silverstein A., Krieger H.P. (1960). Neurologic complications of cardiac surgery. Trans. Am. Neurol. Assoc..

[16] Egerton N., Kay J.H. (1964). Psychological Disturbances Associated with Open Heart Surgery. Br. J. Psychiatry.

[17] Sotaniemi K.A. (1995). Long-term neurologic outcome after cardiac operation. Ann. Thorac. Surg..

[18] Newman M.F., Kirchner J.L., Phillips-Bute B., Gaver V., Grocott H., Jones R.H., Mark D.B., Reves J.G., Blumenthal J.A. (2001). Longitudinal Assessment of Neurocognitive Function after Coronary-Artery Bypass Surgery. N. Engl. J. Med..

[19] Berger M., Terrando N., Smith S.K., Browndyke J.N., Newman M.F., Mathew J.P. (2018). Neurocognitive Function after Cardiac SurgeryFrom Phenotypes to Mechanisms. Anesthesiology.

[20] Zimpfer D., Czerny M., Kilo J., Kasimir M.-T., Madl C., Kramer L., Wieselthaler G.M., Wolner E., Grimm M. (2002). Cognitive deficit after aortic valve replacement. Ann. Thorac. Surg..

[21] Brækken S.K., Reinvang I., Russell D., Brucher R., Svennevig J.L. (1998). Association between intraoperative cerebral microembolic signals and postoperative neuropsychological deficit: Comparison between patients with cardiac valve replacement and patients with coronary artery bypass grafting. J. Neurol. Neurosurg. Psychiatry.

[22] Knipp S.C., Matatko N., Schlamann M., Wilhelm H., Thielmann M., Forsting M., Diener H.C., Jakob H. (2005). Small ischemic brain lesions after cardiac valve replacement detected by diffusion-weighted magnetic resonance imaging: Relation to neurocognitive function. Eur. J. Cardiothorac. Surg..

[23] Gleason T.G., Schindler J.T., Adams D.H., Reardon M.J., Kleiman N.S., Caplan L.R., Conte J.V., Deeb G.M., Hughes G.C., Chenoweth S. (2016). The risk and extent of neurologic events are equivalent for high-risk patients treated with transcatheter or surgical aortic valve replacement. J. Thorac. Cardiovasc. Surg..

[24] Kapadia S.R., Huded C.P., Kodali S.K., Svensson L.G., Tuzcu E.M., Baron S.J., Cohen D.J., Miller D.C., Thourani V.H., Herrmann H.C. (2018). Stroke After Surgical Versus Transfemoral Transcatheter Aortic Valve Replacement in the PARTNER Trial. J. Am. Coll. Cardiol..

[25] Auffret V., Campelo-Parada F., Regueiro A., Del Trigo M., Chiche O., Chamandi C., Allende R., Cordoba-Soriano J.G., Paradis J.-M., De Larochellière R. (2016). Serial Changes in Cognitive Function Following Transcatheter Aortic Valve Replacement. J. Am. Coll. Cardiol..

[26] Srinivasa P., Szerlip M., Al-Azizi K., Harrington K., Kodali S., Kapadia S., Lu M., Leon M.B., Mack M.J. (2020). Neurocognitive Function Change in Low-Risk Patients Undergoing TAVR Versus SAVR. JACC Cardiovasc. Interv..

[27] Reardon M.J., Van Mieghem N.M., Popma J.J., Kleiman N.S., Søndergaard L., Mumtaz M., Adams D.H., Deeb G.M., Maini B., Gada H. (2017). Surgical or Transcatheter Aortic-Valve Replacement in Intermediate-Risk Patients. N. Engl. J. Med..

[28] Leon M.B., Smith C.R., Mack M., Makkar R., Svensson L.G., Kodali S., Thourani V.H., Tuzcu E.M., Miller D.C., Herrmann H.C. (2016). Transcatheter or Surgical Aortic-Valve Replacement in Intermediate-Risk Patients. N. Engl. J. Med..

[29] Abdel-Wahab M., Landt M., Neumann F.J., Massberg S., Frerker C., Kurz T., Kaur J., Toelg R., Sachse S., Jochheim D. (2020). 5-Year Outcomes After TAVR With Balloon-Expandable Versus Self-Expanding Valves: Results From the CHOICE Randomized Clinical Trial. JACC Cardiovasc. Interv..

[30] Agarwal S., Parashar A., Kumbhani D.J., Svensson L.G., Krishnaswamy A., Tuzcu E.M., Kapadia S.R. (2015). Comparative meta-analysis of balloon-expandable and self-expandable valves for transcatheter aortic valve replacement. Int. J. Cardiol..

[31] Durko Andras P., Reardon Michael J., Kleiman Neal S., Popma J.J., Van Mieghem N.M., Gleason T.G., Bajwa T., O’Hair D., Brown D.L., Ryan W.H. (2018). Neurological Complications After Transcatheter Versus Surgical Aortic Valve Replacement in Intermediate-Risk Patients. J. Am. Coll. Cardiol..

[32] Leon Martin B., Mack Michael J., Hahn Rebecca T., Thourani V.H., Makkar R., Kodali S.K., Alu M.C., Madhavan M.V., Chau K.H., Russo M. (2021). Outcomes 2 Years After Transcatheter Aortic Valve Replacement in Patients at Low Surgical Risk. J. Am. Coll. Cardiol..

[33] Phan K., Xie A., Tsai Y.C., Black D., Di Eusanio M., Yan T.D. (2015). Ministernotomy or minithoracotomy for minimally invasive aortic valve replacement: A Bayesian network meta-analysis. Ann. Cardiothorac. Surg..

[34] Blackman D.J., Baxter P.D., Gale C.P., Moat N.E., MacCarthy P.A., Hildick-Smith D., Trivedi U., Cunningham D., De Belder M.A., Ludman P.F. (2013). Do Outcomes from Transcatheter Aortic Valve Implantation Vary According to Access Route and Valve Type? The UK TAVI Registry. J. Interv. Cardiol..

[35] Zhan Y., Saadat S., Soin A., Kawabori M., Chen F.Y. (2019). A meta-analysis comparing transaxillary and transfemoral transcatheter aortic valve replacement. J. Thorac. Dis..

[36] Dahle T.G., Kaneko T., McCabe J.M. (2019). Outcomes Following Subclavian and Axillary Artery Access for Transcatheter Aortic Valve Replacement. JACC Cardiovasc. Interv..

[37] Armijo G., Nombela-Franco L., Tirado-Conte G. (2018). Cerebrovascular Events after Transcatheter Aortic Valve Implantation. Front. Cardiovasc. Med..

[38] Davlouros P.A., Mplani V.C., Koniari I., Tsigkas G., Hahalis G. (2018). Transcatheter aortic valve replacement and stroke: A comprehensive review. J. Geriatr. Cardiol..

[39] Antunes P.E., De Oliveira J.F., Antunes M.J. (2003). Predictors of cerebrovascular events in patients subjected to isolated coronary surgery. The importance of aortic cross-clamping. Eur. J. Cardiothorac. Surg..

[40] Nombela-Franco L., Webb J.G., De Jaegere P.P., Toggweiler S., Nuis R.-J., Dager A.E., Amat-Santos I.J., Cheung A., Ye J., Binder R.K. (2012). Timing, Predictive Factors, and Prognostic Value of Cerebrovascular Events in a Large Cohort of Patients Undergoing Transcatheter Aortic Valve Implantation. Circulation.

[41] Van Mieghem N.M., El Faquir N., Rahhab Z., Rodríguez-Olivares R., Wilschut J., Ouhlous M., Galema T.W., Geleijnse M.L., Kappetein A.-P., Schipper M.E. (2015). Incidence and Predictors of Debris Embolizing to the Brain During Transcatheter Aortic Valve Implantation. JACC Cardiovasc. Interv..

[42] Kroon H., von der Thusen J.H., Ziviello F., van Wiechen M., Ooms J.F., Kardys I., Schipper M., van Gils L., Daemen J., de Jaegere P. (2021). Heterogeneity of debris captured by cerebral embolic protection filters during TAVI. Eurointervention.

[43] Grodecki K., Tamarappoo B.K., Huczek Z., Jedrzejczyk S., Cadet S., Kwiecinski J., Rymuza B., Parma R., Olasinska-Wisniewska A., Fijalkowska J. (2020). Non-calcific aortic tissue quantified from computed tomography angiography improves diagnosis and prognostication of patients referred for transcatheter aortic valve implantation. Eur. Heart J. Cardiovasc. Imaging.

[44] Mack M.J., Acker M.A., Gelijns A.C., Overbey J.R., Parides M.K., Browndyke J.N., Groh M.A., Moskowitz A.J., Jeffries N.O., Ailawadi G. (2017). Effect of Cerebral Embolic Protection Devices on CNS Infarction in Surgical Aortic Valve Replacement: A Randomized Clinical Trial. JAMA.

[45] Kapadia S.R., Kodali S., Makkar R., Mehran R., Lazar R.M., Zivadinov R., Dwyer M.G., Jilaihawi H., Virmani R., Anwaruddin S. (2017). Protection Against Cerebral Embolism During Transcatheter Aortic Valve Replacement. J. Am. Coll. Cardiol..

[46] Seeger J., Gonska B., Otto M., Rottbauer W., Wöhrle J. (2017). Cerebral Embolic Protection During Transcatheter Aortic Valve Replacement Significantly Reduces Death and Stroke Compared with Unprotected Procedures. JACC Cardiovasc. Interv..

[47] Van Mieghem N.M., Van Gils L., Ahmad H., Van Kesteren F., Van Der Werf H.W., Brueren G., Storm M., Lenzen M., Daemen J., van den Heuvel A.F.M. (2016). Filter-based cerebral embolic protection with transcatheter aortic valve implantation: The randomised MISTRAL-C trial. Eurointervention.

[48] Boston Scientific Corporation (2021). PROTECTED TAVR: Stroke PROTECTion With SEntinel During Transcatheter. Clinical Trial Registration NCT04149535, clinicaltrials.gov. NCT04149535.

[49] Kharbanda R. The Role of Cerebral Embolic Protection in Preventing Strokes and Improving other Health Outcomes in Patients Receiving a Replacement Heart Valve. https://www.isrctn.com/ISRCTN16665769?q=&filters=conditionCategory:Circulatory%20System,trialStatus:Ongoing&sort=&offset=1&totalResults=150&page=1&pageSize=10&searchType=basic-search.

[50] Lansky A.J., Schofer J., Tchetche D., Stella P., Pietras C.G., Parise H., Abrams K., Forrest J.K., Cleman M., Reinöhl J. (2015). A prospective randomized evaluation of the TriGuardTM HDH embolic DEFLECTion device during transcatheter aortic valve implantation: Results from the DEFLECT III trial. Eur. Heart J..

[51] Nazif T.M., Moses J., Sharma R., Dhoble A., Rovin J., Brown D., Horwitz P., Makkar R., Stoler R., Forrest J. (2021). Randomized Evaluation of TriGuard 3 Cerebral Embolic Protection After Transcatheter Aortic Valve Replacement: REFLECT II. JACC Cardiovasc. Interv..

[52] Panchal H.B., Paul T.K. (2018). Editorial commentary: Use of cerebral embolic protection devices during transcatheter aortic valve replacement. Trends Cardiovasc. Med..

